# Re-irradiation of recurrent IDH-wildtype glioblastoma in the bevacizumab and immunotherapy era: Target delineation, outcomes and patterns of recurrence

**DOI:** 10.1016/j.ctro.2023.100697

**Published:** 2023-10-30

**Authors:** Sebastian M. Christ, Gilbert Youssef, Shyam K. Tanguturi, Daniel Cagney, Diana Shi, J. Ricardo McFaline-Figueroa, Ugonma Chukwueke, Eudocia Q. Lee, Caroline Hertler, Nicolaus Andratschke, Michael Weller, David A. Reardon, Daphne Haas-Kogan, Matthias Guckenberger, Patrick Y. Wen, Rifaquat Rahman

**Affiliations:** a Department of Radiation Oncology, Dana-Farber/Brigham and Women’s Cancer Center, Harvard Medical School, Boston, MA, USA; b Department of Radiation Oncology, University Hospital and University of Zurich, Zurich, Switzerland; c Center for Neuro-Oncology, Dana-Farber Cancer Institute, Boston, MA, USA; d Competence Center Palliative Care, University Hospital and University of Zurich, Zurich, Switzerland; e Department of Neurology, University Hospital and University of Zurich, Zurich, Switzerland

**Keywords:** Re-irradiation, Recurrent glioblastoma, New systemic therapies

## Abstract

**Introduction and background:**

While recurrent glioblastoma patients are often treated with re-irradiation, there is limited data on the use of re-irradiation in the setting of bevacizumab (BEV), temozolomide (TMZ) re-challenge, or immune checkpoint inhibition (ICI). We describe target delineation in patients with prior anti-angiogenic therapy, assess safety and efficacy of re-irradiation, and evaluate patterns of recurrence.

**Materials and methods:**

Patients with a histologically confirmed diagnosis of glioblastoma treated at a single institution between 2013 and 2021 with re-irradiation were included. Tumor, treatment and clinical data were collected. Logistic and Cox regression analysis were used for statistical analysis.

**Results:**

One hundred and seventeen recurrent glioblastoma patients were identified, receiving 129 courses of re-irradiation. In 66 % (85/129) of cases, patients had prior BEV. In the 80 patients (62 %) with available re-irradiation plans, 20 (25 %) had all T2/FLAIR abnormality included in the gross tumor volume (GTV). Median overall survival (OS) for the cohort was 7.3 months, and median progression-free survival (PFS) was 3.6 months. Acute CTCAE grade ≥ 3 toxicity occurred in 8 % of cases. Concurrent use of TMZ or ICI was not associated with improved OS nor PFS. On multivariable analysis, higher KPS was significantly associated with longer OS (p < 0.01). On subgroup analysis, patients with prior BEV had significantly more marginal recurrences than those without (26 % vs. 13 %, p < 0.01).

**Conclusion:**

Re-irradiation can be safely employed in recurrent glioblastoma patients. Marginal recurrence was more frequent in patients with prior BEV, suggesting a need to consider more inclusive treatment volumes incorporating T2/FLAIR abnormality.

## Highlights

• Data is limited for glioblastoma re-irradiation with BEV, TMZ, and ICI.
• Re-irradiation with concurrent therapy is safe, with little acute CTCAE grade ≥ 3 toxicity.
• Patients with prior BEV have significantly more marginal recurrences.
• Target volume delineation might need to be more inclusive in pretreated patients.

## Introduction and background

Standard treatment for newly diagnosed glioblastoma consists of maximal safe resection followed by chemoradiation and maintenance chemotherapy [1], [2]. Nevertheless, prognosis remains poor, with median time to tumor recurrence of 7–10 months [3]. Recurrence occurs locally in 70–90 % of cases while 10–30 % of relapses occur in a marginal, distant or multifocal fashion. In the recurrent setting, no standard-of-care is established; repeat surgery, re-irradiation (re-RT), clinical trials, systemic therapy (e.g., lomustine, temozolomide (TMZ) re-challenge), tumor-treating fields (TTF), bevacizumab (BEV) or best supportive care can be considered [4], [5]. Owing to technological advances in treatment planning and delivery, re-RT, increasingly in combination with newer systemic therapy agents, can be considered for patients. Several retrospective case series examining re-RT in recurrent glioma patients exist in the literature but do not focus on glioblastomas or the impact of systemic therapies on re-RT [6], [7], [8], [9], [10], [11], [12], [13], [14].

Retrospective case series and RTOG 1205, a phase II study evaluating re-irradiation with or without BEV, have demonstrated that modern, highly conformal re-RT for primary brain tumor relapse can be safely administered in combination with BEV [15]. Despite a lack of overall survival (OS) benefit in phase III trials in newly diagnosed glioblastoma [16], [17] and in recurrent glioblastoma [18], BEV has become a mainstay in the management of recurrent glioblastoma in the US. Its anti-angiogenic effects can help stabilize symptoms, though it can also impact the radiographic appearance of tumors, increasing importance of evaluating T2/FLAIR abnormality in following BEV-treated patients [19]. Pseudo-response and non-enhancement of tumor tissue after BEV exposure also create challenges for re-irradiation given implications of anti-angiogenic effects for target delineation and response assessment; data remains sparse for re-RT in this setting.

The challenge of patient selection remains highly relevant, to better identify patients that may benefit most from re-RT, as well as understanding optimal combinations with systemic therapies. For example, while results with immune checkpoint inhibitors (ICIs) have thus far been disappointing in glioblastoma [20], [21], [22], there has been interest in combining with RT to yield improved immune-mediated responses [23]. As re-RT has recently been administered more frequently in combination with BEV, TMZ re-challenge and ICIs, questions of efficacy and safety, recurrence patterns, and treatment planning are of growing importance.

The aim of this study is to assess the efficacy and safety of re-RT for recurrent glioblastoma in the era of new systemic therapy options. We sought to describe target delineation, assess clinical outcomes and toxicity, identify factors associated with benefit from re-RT and characterize patterns-of-failure in the bevacizumab era.

## Materials and methods

### Study design and patient population

Our study employed a retrospective review from a single, large academic center. The electronic medical record (EMR) was screened for patients with a histologically confirmed diagnosis of glioblastoma. Patients who were seen at our cancer center between 2013 and 2021 were included in this study if they: (1) were 18 years or older at the time of diagnosis; (2) had a glioblastoma diagnosis per the 5th edition of the WHO CNS tumor classification, i.e., an IDH-wildtype tumor [24]; (3) had at least one course of re-irradiation for recurrent or progressive glioblastoma; and (4) had available clinical and/or imaging data available for analysis.

### Data collection

For all patients included in this study, demographic, tumor, treatment, and clinical data were retrieved from the EMR or radiation oncology treatment planning system. Recurrence patterns after initial RT and re-RT were assessed via review of follow-up magnetic resonance imaging (MRI) scans and fusion of RT and re-RT plans when available.

### Treatment planning process

All re-RT patients received MRI with gadolinium of the brain and a computed tomography (CT) in treatment position wearing a thermoplastic fitted mask with a stereotactic image-guided workflow. Enhancing tumor on the T1-weighted MRI sequence was included in the gross tumor volume (GTV) for all patients. In patients who underwent re-resection for recurrence, the GTV comprised the resection cavity and residual tumor. Inclusion of T2-weighted fluid attenuated inversion recovery (FLAIR) abnormalities into the GTV was at the discretion of the treating radiation oncologist. Expansion for the clinical target volume (CTV) was 0–7 mm. The planning target volume (PTV) margin was between 2 and 5 mm. Patients received either fractionated RT (3DCRT/IMRT) or stereotactic regimens (SRS/SRT), employing common re-RT fractionation schedules. Addition of systemic therapies is at the discretion of the treating neuro-oncologists, and concurrent systemic therapy is usually defined as starting within two weeks of re-RT start.

### Study end points and imaging assessment

OS after re-RT was calculated from the start date of re-RT to the date of death. Progression-free survival (PFS) was defined as the time from the start date of re-RT to the date of recurrence/progression. Recurrence on imaging after RT and re-RT was assessed by two independent readers following the Response Assessment in Neuro-Oncology (RANO) criteria for glioma response assessment [25] and clinical notes. For toxicity, we limited the analysis and reporting of acute toxicity to grade 3–5 events (<12 weeks after end of re-RT) according to the Common Terminology Criteria for Adverse Events (CTCAE v.5). Recurrence after initial RT and re-RT was classified into (1) local, (2) marginal [abutting PTV, up to 5 mm to edge], (3) distant [more than 5 mm away from PTV], and (4) multifocal. Patients without follow-up cMRI scans available to document tumor progression after re-RT were censored at the last follow-up encounter.

### Statistical analysis

Appropriate descriptive statistics such as the median and the range were calculated for all variables under study. To calculate OS and PFS, data was coded as time-to-event data and the Kaplan-Meier method was used to analyze and display outcomes. Cox proportional hazard and logistic regression analyses were employed for OS and PFS predictor identification. Selection of potential predictors was done before conducting any analysis and based on the pertinent literature. The proportional hazard assumption was tested employing the Schoenfeld residuals method. For equivalent dose in 2 Gy single fraction (EQD2) calculations, we assumed a tumor alpha/beta ratio of 10 Gy; total EQD2 refers to the summation of the first and second radiation course. Statistical significance was set at p ≤ 0.05. Descriptive and inferential statistics were calculated using the statistical software package STATA® (v.16.0).

### Ethical approval

Institutional review board approval (IRB) for this retrospective single-center cohort study was obtained before project initiation. This project complied with the *World Medical Association International Code of Medical Ethics* and the STROBE checklist for observational cohort studies (see Supplementary Table 1).

## Results

### Study population

Basic patient characteristics are presented in Table 1. The study population consisted of 117 recurrent glioblastoma patients who had received a total of 129 courses of re-irradiation. At time of initial diagnosis, 117 (99 %) patients had a supratentorial tumor, whereas one (1 %) had an infratentorial tumor. The majority (105/117; 95 %) of patients received ≥ 59.4 Gy RT dose with concurrent TMZ. Median age at re-RT was 58 (interquartile range (IQR), 52–64) years; 44 (38 %) patients were female. Median Karnofsky performance status (KPS) at re-RT was 80 (IQR, 80–90). All 117 (100 %) patients included into this study had a histologically confirmed diagnosis of isocitrate dehydrogenase (IDH) wildtype glioblastoma. MGMT promotor methylation status was methylated in 49 (42 %), partially methylated in 7 (6 %), and unmethylated in 46 (39 %) patients; MGMT promotor methylation status assessment could not be performed in 15 (13 %) patients. At diagnosis, more than half of the tumors (73/126; 58 %) were located in the frontal or temporal lobes.

**Table 1:** Patient characteristics.

**Parameter**	**n**^1^	**Data**
Age at re-RT, median (IQR)	129	58 (52–64)
Female sex, n (%)	117	44 (38)
KPS at re-RT, median (IQR)	101	
≥90		35 (35 %)
70–80		60 (59 %)
≤60		6 (6 %)
IDH status, n (%)	117	
Wildtype		117 (1 0 0)
Mutant/unknown		0 (0)
MGMT promotor status, n (%)	117	
Methylated		49 (42)
Partially methylated		7 (6)
Unmethylated		46 (39)
Assessment not performed		15 (13)
Initial tumor location, n (%)	117	
Frontal		24 (21)
Parietal		15 (13)
Temporal		31 (26)
Occipital		11 (9)
Frontoparietal		4 (3)
Frontotemporal		2 (2)
Parietotemporal		11 (9)
Parietooccipital		5 (4)
Occipitotemporal		3 (3)
Thalamical		3 (3)
Corpus callosum		1 (1)
Cerebellar		1 (1)
Multifocal		6 (5)
Type of initial surgery, n (%)	117	
GTR		65 (56)
STR		40 (34)
Biopsy		12 (10)
Initial RT ≥ 59.4 Gy, n (%)	111	105 (95)
Initial RT < 59.4 Gy, n (%)	111	2 (5)
Concurrent TMZ, n (%)^2^	111	105 (95)

*Abbreviations:* GTR = Gross total resection; Gy = Gray; IDH = Isocitrate dehydrogenase; IQR = Interquartile range; KPS = Karnofsky performance status; MGMT = O^6^-methylguanine-DNA methyltransferase; RT = Radiation therapy; STR = Subtotal resection; TMZ = Temozolomide.

1 N = 117 patients who received n = 129 re-RT courses.

2 As part of the initial therapeutic regimen.

The median time to first recurrence was 14.5 (IQR, 8.9–24.1) months. Site of recurrence was local or marginal in 56 % (66/117), distant in 15 % (18/117), and multifocal in 3 % (4/117) of cases. In 22/117 (19 %) cases, the site of tumor relapse was unknown. Almost three fourths of patients had re-RT after the first or second recurrence (90/117; 69 %). Less than 20 % of patients had a tumor resection within six weeks before re-RT (23/117; 17 %), less than half of which resulted in a gross total resection (GTR) (10/23; 45 %), and the remaining were subtotal resection or biopsy (13/23; 55 %).

### Re-RT details

Amongst re-RT patients, 54 % (70/129) had fractionated RT (IMRT/3DCRT) and 29 % (38/129) received SRS/SRT in 1–5 fractions. Median total EQD2, accumulating the first and second radiation dose, was 99.4 Gy (IQR, 99.4–110.8). Median PTV size was 34.1 cm^3^ (IQR, 13.1–105.4). In the 80 patients where a detailed re-RT planning analysis was possible, 20 (25 %) had T2/FLAIR abnormality included into the GTV. Median GTV-to-CTV expansion was 0 mm, with a range of 0 mm to 7 mm. A total of 97 % (127/129) re-RT courses were completed as planned. Re-RT was associated with acute CTCAE grade 3 (seizures, new headaches, subacute infarcts) and 4 (hospitalization for seizures and/or mental status changes, resulting in pausing or canceling of RT) toxicity in 5 % (7/129) and 3 % (4/129) patients, respectively. No acute grade 5 toxicities were observed. Hospitalization rate within three months of end of re-RT was 16 % (20/129).

In 53/129 (41 %) of cases, re-RT was combined with either TMZ (23/129; 18 %) or ICIs (30/129; 23 %) with a PD-1 inhibitor (26/30; 87 %% Pembrolizumab; 4/30; 13 % Nivolumab). Prior to re-RT 66 % (85/129) of patients had received BEV, while 58 % (75/129) received BEV during re-RT. At the start of re-RT, 35 % (45/122) of patients were on dexamethasone, with a median dose of 4 mg (IQR, 2–7); at the end of re-RT, 39 % (50/122) were on dexamethasone, with a median dose of 4 mg (IQR, 2–6).

### Pattern-of-failure after re-RT

Among patients with known pattern-of-failure (80/129; 62 %), site of recurrence after re-RT was local in 27 %, marginal in 22 %, and distant/multifocal in 20 % of cases. Median PFS after re-RT was 3.6 (1.9–5.1) months (see Fig. 1a). At six months, 16 % (17/117) of patients were progression-free. Median OS after re-RT was 7.3 (4.3–11.0) months (see Fig. 1b). Within 90 days after end of re-RT, 10 % (12/117) patients died. Upon progression, roughly one third of patients was transferred to hospice care (34/129; 26 %). Table 2 summarizes management at recurrence after re-RT.

**Fig. 1a:**
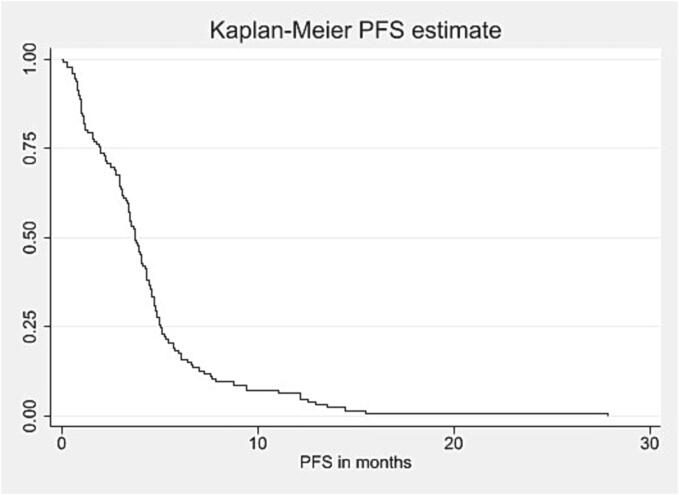
Kaplan-Meier curve for PFS after re-RT in months*, *Abbreviations:* PFS = Progression-free survival; Re-RT = Reirradiation. *Calculated from the start date of re-RT.

**Fig. 1b:**
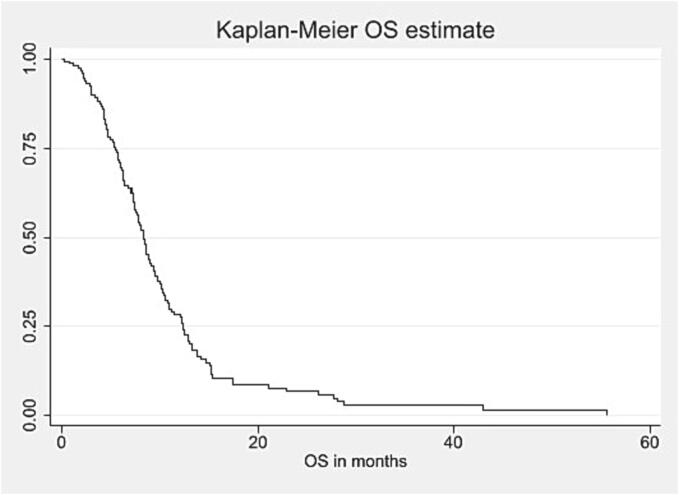
Kaplan-Meier curve for OS after re-RT in months*, *Abbreviations:* OS = Overall survival; Re-RT = Reirradiation. *Calculated from the start date of re-RT.

**Table 2:** Re-irradiation details and therapeutic regimens.

Parameter	**n**^1^	**Data**
Time to recurrence in months^2^, median (IQR)	117	14.5 (8.9–24.1)
Site of recurrence to be re-irradiated after upfront RT, n (%)	117	
Local failure		66 (56)
Marginal failure		7 (6)
Distant failure		18 (15)
Multifocal failure		4 (3)
Unknown		22 (19)
Number of recurrences before re-RT, n (%)	129	
1		47 (36)
2		43 (33)
3		26 (20)
4		7 (5)
≥5		6 (5)
Surgery within 6 weeks before re-RT, n (%)	129	23 (17)
Type of surgery, n (%)	23	
GTR		10 (45)
STR/biopsy		13 (55)
Number of surgeries after initial resection before re-RT, n (%)	129	
0		80 (62)
1		28 (22)
2		18 (14)
≥3		3 (2)
Fractionation schedule, n (%)	129	
18–20 Gy/1 fraction (SRS)		16 (12)
30 Gy/5 fractions (SRT)		22 (17)
35 Gy/10 fractions		50 (39)
37.5 Gy/15 fractions		3 (2)
40.05 Gy/15 fractions		17 (13)
Other		21 (16)
Total EQD2 [1st + 2nd radiation treatment], median (range)	129	99.4 (99.4–110.8)
PTV in cm, median (IQR)	92	34.1 (13.1–105.4)
Inclusion of FLAIR abnormality into GTV definition, n (%)	80	20 (25)
CTV expansion in mm, median (range)	80	0 (0–7)
Completion of re-RT, n (%)	129	127 (98)
Grade 3–5 CTCAEv.5 toxicity, n (%)	129	
Grade 3		7 (5)
Grade 4		4 (3)
Grade 5		0 (0)
Concurrent temozolomide with re-RT, n (%)	129	23 (18)
Concurrent ICI with re-RT, n (%)	129	30 (23)
Prior BEV exposure, n (%)	129	85 (66)
Concurrent BEV with re-RT, n (%)	129	75 (58)
Steroid use at time of re-RT start, n (%)	122	45 (35)
Steroid dose (mg) at time of re-RT start, median (IQR)	122	4 (2–7)
Steroid use at time of re-RT end, n (%)	122	50 (39)
Steroid dose (mg) at time of re-RT end, n (%)	122	4 (2–6)
Hospitalization within 90 days of re-RT, n (%)	129	20 (16)
Site of recurrence after re-RT, n (%)	129	
Local failure		35 (27)
Marginal failure		28 (22)
Distant/multifocal failure		26 (20)
Unknown		40 (31)
PFS after re-RT in months^2^, median (IQR)	129	3.6 (1.9–5.1)
Evidence of radionecrosis after re-RT, n (%)^3^	120	0 (0)
Progression-free patients at 6 months, %	107	17 (16)
Treatment upon progression after re-RT	129	
TMZ +/- BEV		4 (3)
CCNU/BCNU/other +/- BEV		27 (21)
BEV		29 (22)
Hospice		34 (26)
Unknown		35 (27)
OS after re-RT in months^2^, median (IQR)	117	7.3 (4.3–11.0)
Death within 90 days of end of re-RT, n (%)	117	12 (10)

*Abbreviations:* 3DCRT = 3-dimensional conformal radiotherapy; CTCAE = Common Terminology Criteria for Adverse Events; CTV = Clinical target volume; FLAIR = Fluid attenuated inversion recovery; GTR = Gross total resection; GTV = Gross tumor volume; ICI = Immune checkpoint inhibition; IQR = Interquartile range; PTV = Planning target volume; Re-RT = Reirradiation; RT = Radiation therapy; SRS = Stereotactic radiosurgery; SRT = Stereotactic radiotherapy; STR = Subtotal resection.

1 N = 117 patients who received n = 129 re-RT courses.

2 Calculated from the time of initial diagnosis.

3 Short follow-up period and PFS, and limited availability of follow-up brain scans.

### Re-RT amongst patients with prior BEV exposure

Table 3 shows the comparative overview or re-RT with and without prior BEV exposure. When comparing the subgroups of patients who received prior BEV (85/129; 66 %) and those without prior BEV exposure (44/126; 34 %), patients without BEV exposure prior to re-RT were more often re-irradiated on the first tumor recurrence (64 % vs. 24 %) or had surgery within six weeks before re-RT (27 % vs. 12 %). Patients with prior BEV exposure had significantly more marginal recurrences after re-RT compared to patients without prior BEV therapy (26 % vs. 13 %). The inclusion of T2/FLAIR abnormalities into the GTV definition was 36 % in the prior BEV group and 28 % in the BEV-naïve group. In the group of patients with prior BEV exposure who had a marginal recurrence after re-RT, FLAIR abnormalities were included into the GTV definition in only a minority of patients (5/22; 23 %). Moreover, the subgroup of patients who received BEV prior to re-RT compared to those who did not, had a lower OS (7.2 vs. 8.5 months; p < 0.05), but no difference in PFS (3.4 vs. 3.7 months; p = 0.130).

**Table 3:** Comparative overview of re-RT with and without prior BEV exposure.

Parameter	**Prior BEV** **(n = 85)**	**No prior BEV** **(n = 44)**
Age at re-RT, n (%)		
<70	75 (88)	40 (91)
≥70	10 (12)	4 (9)
Gender, n (%)		
Male	53 (62)	29 (66)
Female	32 (38)	15 (34)
KPS at re-RT, n (%)		
≥70	78 (92)	30 (91)
<70^1^	7 (8)	3 (9)
MGMT status, n (%)		
Methylated	30 (40)	18 (51)
Partially methylated/unmethylated^1^	45 (60)	17 (49)
Type of initial surgery, n (%)		
GTR	10 (12)	3 (7)
STR/biopsy	75 (88)	42 (93)
Time to re-RT in months^2^, n (%)		
<16.3 *[median]*	42 (49)	29 (59)
≥16.4	43 (51)	18 (41)
Number of recurrences before re-RT, n (%)		
1	20 (24)	29 (64)
≥2	65 (76)	16 (36)
Surgery within 6 weeks before re-RT, n (%)		
Yes	10 (12)	12 (27)
No	75 (88)	32 (73)
Inclusion of FLAIR into GTV definition, n (%)		
Yes	15 (28)	5 (36)
No^1^	38 (72)	17 (64)
Fraction schedule and re-RT technique, n (%)		
SRS/SRT	20 (34)	9 (38)
10–15 fractions^3^	39 (66)	15 (63)
Type of recurrence after re-RT, n (%)		
Local	17 (20)	17 (38)
Marginal	22 (26)	6 (13)
Distant/multifocal	16 (19)	7 (16)
Unknown	30 (35)	15 (33)

*Abbreviations:* FLAIR = Fluid attenuated inversion recovery; GTR = Gross total resection; GTV = Gross tumor volume; IDH = Isocitrate dehydrogenase; IMRT/VMAT = Intensity modulated radiotherapy/Volumetric Modulated Arc Therapy; IQR = Interquartile range; KPS = Karnofsky performance score; MGMT = O^6^-methylguanine-DNA methyltransferase; Re-RT = Reirradiation; RT = Radiation therapy; SRS = Stereotactic radiosurgery; SRT = Stereotactic radiotherapy; STR = Subtotal resection; TMZ = Temozolomide.

1 Exclusion of “unknown” category.

2 Calculated as time from primary diagnosis until the time of re-RT start.

3 Exclusion of all other fractionation schedules.

### Systemic therapy with re-RT

When assessing OS and PFS in the subgroups of patients who received systemic therapy, there was no statistically significant difference in the use of concurrent TMZ, IO and/or BEV with re-RT relative to patients without. On univariable Cox regression analysis, higher KPS (≥70) (hazard ratio (HR): 0.522; (95 % confidence interval (CI), 0.276–0.923); re-RT at time of first recurrence (HR: 1.430; 95 % CI, 0.786–1.671); and no exposure to BEV prior to re-RT (HR: 1.453; 95 % CI, 1.091–2.234) were associated with longer OS. On multivariable Cox regression analysis, only KPS was statistically significantly associated with OS (<70 vs. ≥ 70; HR: 0.543; 95 % CI, 0.281–0.963) (Supplementary Table 2).

## Discussion

Re-RT is a commonly used treatment option in the setting of modern systemic therapies including BEV, TMZ and IO. Prior BEV exposure often complicates treatment planning due to decreased contrast uptake and possible increase in T2/FLAIR, which was reflected by the observation that patients with prior BEV exposure had significantly more marginal recurrences than those without BEV exposure in this patient series (26 % vs. 13 %). This raises the question of whether all T2/FLAIR abnormalities should be included into the target volume definition in these patients. Re-RT is safe in patients receiving modern systemic therapies, with a prevalence of acute grade 3/4 toxicity of 8 %, and no radiation necrosis observed in this patient cohort. There was no evidence that the addition of TMZ, BEV or ICI to re-RT increases OS or PFS, yet this is a retrospective analysis with a limited sample size, so further clinical studies are required to ascertain this data.

Administration of BEV decreases peritumoral edema and improves neurologic symptoms in glioblastoma patients [26]. BEV may also be helpful to mitigate possible toxicity from radiation-induced edema. While it remains an unsettled question of whether the addition of BEV affects the efficacy of radiation, the altered radiological appearance of tumor after BEV exposure has been well-documented [27]. Traditionally, contouring re-RT for recurrent glioblastoma has focused on enhancing tumor only [12]. Given that there also can be non-enhancing tumor progression particularly among patients with prior BEV exposure, however, targeting enhancement alone may not be sufficient in select patient populations.

**Fig. 2:**
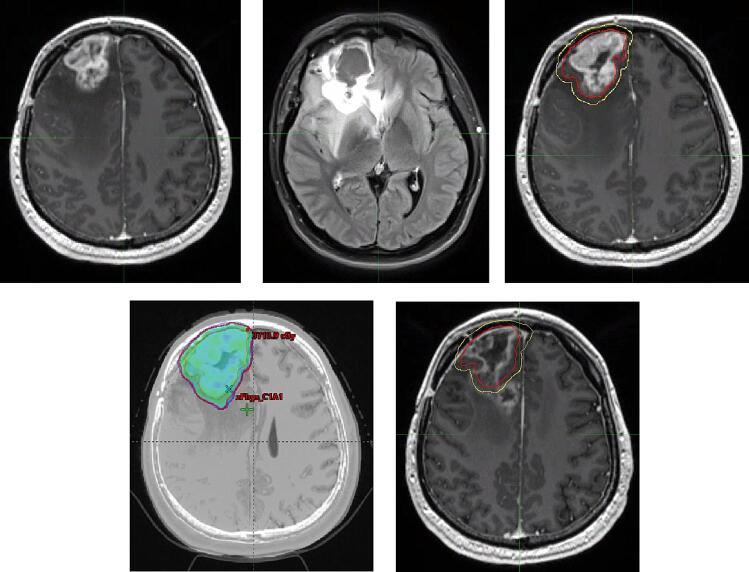
Case example of a marginal recurrence after re-RT. This 29-year-old male was re-irradiated for a right frontal recurrence of a glioblastoma about 10 months after initial diagnosis and treatment. At the time of re-treatment, the patient had already received Bevacizumab. (A) and (B) show 3D AX T1 GAD and 3D AX T2 FLAIR images at recurrence, respectively. The patient was then re-irradiated with 35 Gy/10fx concurrent with Bevacizumab and immune checkpoint inhibition. (C) shows the PTV and CTV contours, (D) shows the 95 % isodose line of IMRT re-irradiation plan. Ten months after re-RT, the patient relapsed again. The progressive, non-nodular enhancement on (E) 3D AX T1 Gad was abutting the re-RT PTV and considered a “marginal recurrence” in this study.

Here we systematically evaluated patterns of recurrence after re-RT in BEV-treated versus BEV-naïve patients. Our results indicate that patients with prior BEV exposure had significantly more frequent marginal recurrences than those without BEV exposure, and only a minority of these patients had all T2/FLAIR abnormalities included into their target volume definition. We appreciate that target volume definition and treatment planning in the recurrent setting can be complicated, and this is true even more so for patients with prior BEV exposure (case example in Fig. 2). PET imaging is not currently a part of routine clinical practice at our institution, but there is emerging evidence that PET imaging might be advantageous in target volume delineation in recurrent glioblastoma [28]. Nonetheless, our results suggest that there is a need for a more generous approach to including T2/FLAIR abnormalities into the target volume definition, which could reduce the rate of marginal recurrences.

Our study confirmed that re-RT is generally safe in selected recurrent glioblastoma patients. Overall, grade 3 and 4 acute AEs occurred in 5 % and 3 % respectively and in up to 15 % (3/20) when the T2/FLAIR abnormality was included into the target volume definition. No acute grade 5 toxicities were observed. Even after a median total EQD2 of 99.4 Gy, no evidence of radiation necrosis was identified in any re-irradiated patient, yet this finding might be due to the median PFS of 3.6 months and small PTV volumes. Toxicity in our series aligned with previously reported data, and they support implementation of re-RT with systemic therapies including ICIs. *Ehret et al. (2023)* observed good tolerability and no high grade toxicity or radiation necrosis after re-RT with a total median EQD2 107.6 Gy [29]. *Kaul et al. (2021)* reported grade 3 toxicity of 5.1 %, grade 4 toxicity of 2.5 %, and grade 5 toxicity of 0.0 % [6]. *Adachi et al. (2019)* observed acute grade 3 or higher toxicity in 11 % of patients [30]. *Baehr et al. (2019)* reported acute toxicity of grade 3 for 3/46 (6.5 %) patients after normofractionated re-RT [31].

In this cohort, the subgroup of patients who received BEV prior to re-RT compared to those who did not have a lower OS (7.2 vs. 8.5 months; p < 0.05), but no difference in PFS (3.4 vs. 3.7 months; p = 0.130). *Tsien et al. (2022)* recently reported the results from the phase II RTOG1205 trial, which showed no difference in OS from adding re-RT to BEV [32]. The difference between their findings and ours may reflect differences in the study populations. While RTOG1205 found an improved 6-months PFS in the concurrent BEV arm [32], PFS after re-RT was not statistically significant between the groups who did and did not have prior BEV exposure in our patient cohort.

There is limited data on patterns-of-failure after re-irradiation. We noted that patients with prior BEV exposure had a higher risk of marginal recurrence compared to patients without prior BEV exposure. We also noted that site of recurrence after re-RT was local in 27 %, marginal in 22 %, and distant/multifocal in 20 % of cases. A smaller study of 23 patients by *Shields et al. (2013)* indicated that most failures were out-of-field [33].

This study represents a large and comprehensive retrospective series on re-irradiated recurrent glioblastoma patients. Our OS and PFS data are comparable to those seen in other recent retrospective series. Very recently, *Ehret et al. (2023)* assessed 88 patients with IDH-wildtype glioblastoma who had undergone re-RT and reported a median OS of 8.0 months as well as a median PFS of 5.9 months [29]. *Kaul et al. (2020)* analyzed 198 patients with gliomas including 133 recurrent and 19 secondary glioblastoma patients, for which a median OS of 6.0 months was reported. Data on PFS and MGMT status was only available for a minority of patients or not reported [6]. *Adachi et al. (2019)* published data on 35 glioma patients, 20 of which had robotic SRT for recurrent glioblastoma and showed a median OS and PFS after re-RT of 9.0 and 3.0 months, respectively [30]. *Sallabanda et al. (2019)*, *Yaprak et al. (2019)*, and *Gigliotti et al. (2019)* reported median OS for highly selected groups of 24, 42 and 25 glioblastoma patients, who had received stereotactic re-RT, of 8.0, 12.0, and 9.0 months [34], [35], [36]. Our study thus confirms that re-RT is a viable option for selected patients with acceptable levels of toxicity in setting of systemic therapies such as BEV, ICIs, and TMZ rechallenge. Prospective data will be important to better delineate the efficacy of re-RT in combination with new systemic therapies, and there are several ongoing efforts (e.g., NCT03782415, NCT05463848, NCT04729959, NCT03532295, and NCT04145115).

With re-RT being a safe and viable option for patients with recurrent glioblastoma, the most pressing question is patient selection to identify those who may be most likely to benefit from repeat local therapy. Some authors have found evidence for benefit of re-resection in the recurrent setting [29], [37]. Re-resection was not a statistically significant predictor in our cohort, but further study is warranted to better understand and identify patients who may benefit from re-resection, re-irradiation or both.. In fact, of all predictor variables examined here, only higher KPS was statistically significant, which is consistent with several other studies. Other studies have provided conflicting results on the concurrent use of TMZ during re-RT. *Baehr et al. (2019)* and *Grosu et al. (2005)* found a positive association of concurrent TMZ with clinical outcomes in all and MGMT promoter methylated patients, respectively [31], [38]. In their study of 147 glioma patients, *Fogh et al. (2010)* reported no OS benefit from chemotherapy including TMZ, similar to our study [11]. The subgroup of patients with concurrent ICI and re-RT had no difference in OS or PFS compared to those who did not. but conclusions are limited by small numbers of patients and variations in therapy administration.

Conclusions from our study are subject to several limitations including the retrospective nature of patient review and the highly selected patient cohort. The study population may not be representative since the initial PFS of > 15 months exceeds population-level estimates. In addition, while we reviewed all available plans, RT plans of patients treated at an external institution were not available for review. Nonetheless, we have attempted to reduce variability in the patient population by excluding known IDH mutant tumors in line with the 2021 WHO classification schema.

In conclusion, our study confirms that re-RT in glioblastoma patients is a safe option in the recurrent setting for select patients, including patients with prior BEV exposure. Concurrent TMZ and ICI did not show a clear benefit in this cohort but further study is required to better evaluate possible complementary benefits of systemic therapies with re-RT. Marginal recurrence was significantly more frequent in patients who had prior BEV exposure, highlighting the importance of including T2/FLAIR abnormalities into target volumes when safe to do so.

## Ethics approval

This study was approved by the Institutional Review Board of DFCI/BWH before the initiation of the project (IRB #13-140).

## Availability of data and material

Collected patient and imaging data are confidential and not available for publication.

## Code availability

Not applicable for this publication.

## Authors’ contributions

All authors made significant contributions to this project.

## Conflicts of interest/Competing interests

CH has received research support through the “Filling the Gap” Program of the University of Zurich. CH has received honoraria from Vifor. DAR has received research support through DFCI from Acerta Phamaceuticals; Agenus; Bristol-Myers Squibb; Celldex; EMD Serono; Enterome; Epitopoietic Research Corporation; Incyte; Inovio; Insightec; Merck; Novartis; Omniox; Tragara. DAR has received advisory fees from Agios; AnHeart Therapeutics; Avita Biomedical, Inc., Bristol Myers Squibb; Boston Biomedical; CureVac AG; Del Mar Pharma; DNAtrix; Hoffman-LaRoche, Ltd; Imvax; Janssen; Kiyatec; Medicenna Therapeutics; Neuvogen; Novartis; Novocure; Pyramid; Sumitomo Dainippon Pharma; Vivacitas Oncology, Inc Y-mabs Therapeutics. DAR has received Honoraria from Abbvie; Advantagene; Agenus; Agios; Amgen; Avita Biomedical, Inc., Bayer; Boston Biomedical; Boehringer Ingelheim; Bristol-Myers Squibb; Celldex; Deciphera; DelMar; Ellipses Pharma; EMD Serono; Genenta; Genentech/Roche; Imvax; Inovio; Kintara; Kiyatec; Medicenna Biopharma, Inc.; Merck; Merck KGaA; Monteris; Neuvogen; Novartis; Novocure; Oncorus; Oxigene; Regeneron; Stemline; Sumitono Dainippon Pharma; Taiho Oncology, Inc. NA reports personal fees from AstraZeneca; Debiopharm; ViewRay; BrainLab. NA received research grants from ViewRay. MG has received research support from ViewRay; AstraZeneca. MG is the current President-elect of ESTRO. MW has received research grants from Versameb; Quercis. MW has received consulting fees from Bayer; Curevac; Medac; Novocure; Philogen; Roche; Sandoz. MW has received honoraria from Novartis. MV holds a board role at Orbus. PY has received research grants from Astra Zeneca/Medimmune; Black Diamond; Beigene; Celgene; Chimerix; Eli Lily; Erasca; Genentech/Roche; Kazia; MediciNova; Merck; Novartis; Nuvation Bio; Puma, Servier; Vascular Biogenics; VBI Vaccines. PY has received consulting fees from Astra Zeneca, Bayer; Black Diamond; Boehringer Ingelheim; Boston Pharmaceuticals; Celularity; Chimerix; Day One Bio; Genenta; Glaxo Smith Kline; Karyopharm; Merck, Mundipharma; Novartis; Novocure; Nuvation Bio; Prelude Therapeutics; Sapience; Servier; Sagimet; Vascular Biogenics; VBI Vaccines. PY holds board positions at Novocure; Day One Bio.

## Funding

SMC received support through the “Young Talents in Clinical Research” Beginner’s Grant from the Swiss Academy of Medical Sciences (SAMW) and the Bangerter-Rhyner Foundation.

## Declaration of Competing Interest

The authors declare that they have no known competing financial interests or personal relationships that could have appeared to influence the work reported in this paper.
