# Supplementary material for: Re-irradiation of recurrent IDH-wildtype glioblastoma in the bevacizumab and immunotherapy era: Target delineation, outcomes and patterns of recurrence
Source: Clin Transl Radiat Oncol. 2023 Oct 30;44:100697. doi: 10.1016/j.ctro.2023.100697 (PMC10689476; doi:10.1016/j.ctro.2023.100697)
Supplement: Supplementary data 1 [file mmc1.docx]

Supplementary table 1: STROBE checklist

|  | Item No. | Recommendation | Relevant text from manuscript |
| --- | --- | --- | --- |
| **Title and abstract** | 1 | (a) Indicate the study’s design with a commonly used term in the title or the abstract | Single institution, retrospective patient cohort analysis regarding the re-irradiation of recurrent IDH-wildtype glioblastoma in the bevacizumab and immunotherapy era: Target delineation, outcomes and patterns of recurrence. |
|  |  | (b) Provide in the abstract an informative and balanced summary of what was done and what was found | One hundred and thirty-six recurrent glioblastoma patients who received 129 courses of fractionated or stereotactic re-irradiation for recurrent glioblastoma were identified and analyzed. We found that re-irradiation is safe. Marginal recurrence was more frequent in patients who had prior BEV exposure, suggesting a need to consider more inclusive treatment volumes incorporating T2/FLAIR abnormality given known differences in patterns of progressive disease in these patients. |
| Introduction | |  |  |
| Background/rationale | 2 | Explain the scientific background and rationale for the investigation being reported | Re-RT is a viable option in the recurrent glioblastoma setting, as little has changed in the therapeutic landscape for this dismal disease, which remains associated with a terrible prognosis. As re-RT has recently been administered more frequently in combination with BEV, TMZ re-challenge and IO, questions of treatment planning, efficacy and safety, and recurrence patterns are of growing importance. The challenge of patient selection therefore remains highly relevant, to better identify which patients which benefit most from re-RT and understanding optimal combinations with systemic therapies. |
| Objectives | 3 | State specific objectives, including any prespecified hypotheses | The aim of this study was to assess the efficacy and safety of re-RT for recurrent glioblastoma in the era of new systemic therapy options, including BEV and ICI. We sought to describe target delineation, assess clinical outcomes and toxicity, identify factors associated with benefit from re-RT and characterize patterns-of-failure in the setting of prior BEV exposure. |
| Methods | |  |  |
| Study design | 4 | Present key elements of study design early in the paper | This study was designed as a retrospective single-center cohort study at large academic medical center. The electronic medical record (EMR) was screened for patients with a histologically confirmed diagnosis of IDH wildtype glioblastoma. Patients who were seen at our cancer center between 2013-2021 were included in this study if they (1) were 18 years or older at the time of diagnosis, (2) had glioblastoma, and (3) had at least one course of re-irradiation for recurrent or progressive glioblastoma, and (4) had available clinical and imaging data to allow for analysis. |
| Setting | 5 | Describe the setting, locations, and relevant dates, including periods of recruitment, exposure, follow-up, and data collection |  |
| Participants | 6 | (a) Cohort study—Give the eligibility criteria, and the sources and methods of selection of participants. Describe methods of follow-up  Case-control study—Give the eligibility criteria, and the sources and methods of case ascertainment and control selection. Give the rationale for the choice of cases and controls  Cross-sectional study—Give the eligibility criteria, and the sources and methods of selection of participants |  |
|  |  | (b) Cohort study—For matched studies, give matching criteria and number of exposed and unexposed  Case-control study—For matched studies, give matching criteria and the number of controls per case | N/a. |
| Variables | 7 | Clearly define all outcomes, exposures, predictors, potential confounders, and effect modifiers. Give diagnostic criteria, if applicable | OS after re-RT was calculated from the start date of re-RT to the date of death. PFS was defined as the time from the start date of re-RT to the date of recurrence/progression. Recurrence on imaging after RT and re-RT was assessed following the Response Assessment in Neuro-Oncology (RANO) criteria for glioma response assessment and clinical notes. For toxicity, we limited the analysis and reporting of acute toxicity to grade 3–5 events (<12 weeks after end of re-RT) according to the Common Terminology Criteria for Adverse Events (CTCAE v.5). Recurrence after initial RT and re-RT was classified into (1) local, (2) marginal [abutting PTV, up to 5mm from the outside], (3) distant, and (4) multifocal. Patients without follow-up cMRI scans available to document tumor progression after re-RT were censored. |
| Data sources/ measurement | 8* | For each variable of interest, give sources of data and details of methods of assessment (measurement). Describe comparability of assessment methods if there is more than one group |  |
| Bias | 9 | Describe any efforts to address potential sources of bias | We applied methodology commonly used in retrospective patient series; selection bias cannot be systematically addressed, unfortunately, yet we explicitly stated this in the shortcomings/limitations in the discussion section. |
| Study size | 10 | Explain how the study size was arrived at | By screening all patients who received a re-RT for recurrent glioblastoma at the institution this study was undertaken at, and by adhering to the above stated inclusion criteria. |
| Quantitative variables | 11 | Explain how quantitative variables were handled in the analyses. If applicable, describe which groupings were chosen and why | Groups were chosen against the backdrop of currently ongoing scientific debates surrounding systemic therapies such as BEV, TMZ or ICI. |
| Statistical methods | 12 | (a) Describe all statistical methods, including those used to control for confounding | Appropriate descriptive statistics were calculated for all variables under study. To calculate OS and PFS, the Kaplan-Meier method was used. Cox proportional hazard and logistic regression analyses were employed for OS and PFS predictor identification. Descriptive and inferential statistics were calculated using the statistical software package STATA® (v.16.0). |
|  |  | (b) Describe any methods used to examine subgroups and interactions |  |
|  |  | (c) Explain how missing data were addressed | The manuscript highlights what data is missing for what patients, including in the tables provided. Patients with missing data/variables were not included into the respective sub-analyses. We also included many tables in the supplement, so that sub-analyses and patient numbers can be clearly followed/understood. |
|  |  | (d) Cohort study—If applicable, explain how loss to follow-up was addressed  Case-control study—If applicable, explain how matching of cases and controls was addressed  Cross-sectional study—If applicable, describe analytical methods taking account of sampling strategy | OS and PFS follow-up data were available for the large majority of patients, for whom radiation plan were available for assessment. When conducting OS and PFS analysis, loss to follow-up was addressed as is typical in the scientific medical literature and retrospective patient series. |
|  |  | (e) Describe any sensitivity analyses | N/a. |
| **Results** |  |  |  |
| Participants | 13* | (a) Report numbers of individuals at each stage of study—eg numbers potentially eligible, examined for eligibility, confirmed eligible, included in the study, completing follow-up, and analyzed | The study population consisted of 117 recurrent glioblastoma patients who had received a total of 129 courses of re-irradiation. All patients and re-RT courses were generally included into the assessment, if data was available. |
|  |  | (b) Give reasons for non-participation at each stage |  |
|  |  | (c) Consider use of a flow diagram | N/a. |
| Descriptive data | 14* | (a) Give characteristics of study participants (eg demographic, clinical, social) and information on exposures and potential confounders | Basic patient characteristics are presented in Table 1. The study population consisted of 117 recurrent glioblastoma patients who had received a total of 129 courses of re-irradiation. At time of initial diagnosis, 117 (99%) patients had a supratentorial tumor, whereas one (1%) had an infratentorial tumor. The majority (105/117; 95%) of patients received ≥59.4Gy RT dose with concurrent TMZ. Median age at re-RT was 58 (interquartile range (IQR), 52–64) years; 44 (38%) patients were female. Median Karnofsky performance status (KPS) at re-RT was 80 (IQR, 80–90). All 117 (100%) patients included into this study had a histologically confirmed diagnosis of isocitrate dehydrogenase (IDH) wildtype glioblastoma. MGMT promotor methylation status was methylated in 49 (42%), partially methylated in 7 (6%), and unmethylated in 46 (39%) patients; MGMT promotor methylation status assessment could not be performed in 15 (13%) patients. At diagnosis, more than half of the tumors (73/126; 58%) were located in the frontal or temporal lobes. |
|  |  | (b) Indicate number of participants with missing data for each variable of interest | Consult manuscript Results section as well as tables in the supplements, where this is displayed in detail. This was especially relevant for patients re-irradiated externally. |
|  |  | (c) Cohort study—Summarize follow-up time (eg, average and total amount) | The median time to first recurrence was 14.5 (IQR, 8.9–24.1) months. Site of recurrence was local or marginal in 56% (66/117), distant in 15% (18/117), and multifocal in 3% (4/117) of cases. In 22/117 (19%) cases, the site of tumor relapse was unknown. Almost three fourths of patients had re-RT after the first or second recurrence (90/117; 69%). Less than 20% of patients had a tumor resection within six weeks before re-RT (23/117; 17%), less than half of which declared a GTR (10/23; 45%), the rest a STR (13/23; 55%). |
| Outcome data | 15* | Cohort study—Report numbers of outcome events or summary measures over time | Amongst re-RT patients, 54% (70/129) had fractionated RT (IMRT/3DCRT) and 29% (38/129) received SRS/SRT in 1-5 fractions. Median total equivalent dose in 2 Gy single fractions (EQD2), accumulating the first and second radiation dose, was 99.4 Gy (IQR, 99.4–110.8). Median PTV size was 34.1 cm3 (IQR, 13.1–105.4). In the 80 patients where a detailed re-RT planning analysis was possible, 20 (25%) had T2/FLAIR abnormality included into the GTV. Median GTV-to-CTV expansion was 0 mm, with a range of 0 mm to 7 mm. A total of 97% (127/129) re-RT courses were completed as planned. Re-RT was associated with acute CTCAE grade 3 (seizures, new headaches, subacute infarcts) and 4 (hospitalization for seizures and/or mental status changes, resulting in pausing or canceling of RT) toxicity in 5% (7/129) and 3% (4/129) patients, respectively. No acute grade 5 toxicities were observed. Hospitalization rate within three months of end of re-RT was 16% (20/129). |
|  |  | Case-control study—Report numbers in each exposure category, or summary measures of exposure |  |
|  |  | Cross-sectional study—Report numbers of outcome events or summary measures |  |
| Main results | 16 | (a) Give unadjusted estimates and, if applicable, confounder-adjusted estimates and their precision (eg, 95% confidence interval). Make clear which confounders were adjusted for and why they were included | Among patients with known pattern-of-failure (80/129; 62%), site of recurrence after re-RT was local in 27%, marginal in 22%, and distant/multifocal in 20% of cases. Median PFS after re-RT was 3.6 (1.9–5.1) months (see Figure 1a). At six months, 16% (17/117) of patients were progression-free. Median OS after re-RT was 7.3 (4.3–11.0) months (see Figure 1b). Within 90 days after end of re-RT, 10% (12/117) patients died. Upon progression, roughly one third of patients was transferred to hospice care (34/129; 26%). Table 2 summarizes management at recurrence after re-RT.  Table 3 shows the comparative overview or re-RT with and without prior BEV exposure. When comparing the subgroups of patients who received prior BEV (85/129; 66%) and those without prior BEV exposure (44/126; 34%), patients without BEV exposure prior to re-RT were more often re-irradiated on the first tumor recurrence (64% vs. 24%) or had surgery within six weeks before re-RT (27% vs. 12%). Patients with prior BEV exposure had significantly more marginal recurrences after re-RT compared to patients without prior BEV therapy (26% vs. 13%). The inclusion of T2/FLAIR abnormalities into the GTV definition was 36% in the prior BEV group and 28% in the BEV-naïve group. In the group of patients with prior BEV exposure who had a marginal recurrence after re-RT, FLAIR abnormalities were included into the GTV definition in only a minority of patients (5/22; 23%). Moreover, the subgroup of patients who received BEV prior to re-RT compared to those who did not, had a lower OS (7.2 vs. 8.5 months; p<0.05), but no difference in PFS (3.4 vs. 3.7 months; p=0.130). |
|  |  | (b) Report category boundaries when continuous variables were categorized |  |
|  |  | (c) If relevant, consider translating estimates of relative risk into absolute risk for a meaningful time period |  |
| Other analyses | 17 | Report other analyses done—eg analyses of subgroups and interactions, and sensitivity analyses | When assessing OS and PFS in the subgroups of patients who received systemic therapy, there was no statistically significant difference in the use of concurrent TMZ, IO and/or BEV with re-RT relative to patients without. |
| **Discussion** |  |  |  |
| Key results | 18 | Summarize key results with reference to study objectives | Re-RT is a commonly used treatment option in the setting of modern systemic therapies including BEV, TMZ and IO. Prior BEV exposure often complicates treatment planning due to decreased contrast uptake and possible increase in T2/FLAIR, which was reflected by the observation that patients with prior BEV exposure had significantly more marginal recurrences than those without BEV exposure in this patient series (26% vs. 13%). This raises the question of whether all T2/FLAIR abnormalities should be included into the target volume definition in these patients. Re-RT is safe in patients receiving modern systemic therapies, with a prevalence of acute grade 3/4 toxicity of 8%, and no radiation necrosis observed in this patient cohort. There was no evidence that the addition of TMZ, BEV or ICI to re-RT increases OS or PFS, yet this is a retrospective analysis with a limited sample size, so further clinical studies are required to ascertain this data. |
| Limitations | 19 | Discuss limitations of the study, taking into account sources of potential bias or imprecision. Discuss both direction and magnitude of any potential bias | Limitations of this study include inherent limitations due to its retrospective nature. An additional challenge was missing data, such as patients who received re-irradiation at an external institution where RT plans were not able to be reviewed. |
| Interpretation | 20 | Give a cautious overall interpretation of results considering objectives, limitations, multiplicity of analyses, results from similar studies, and other relevant evidence | Our study confirms that re-RT in glioblastoma patients is safe and a valid option in the recurrent setting for select patients, including patients with prior BEV exposure. Concurrent TMZ and IO did not show a clear benefit in this cohort but further study is required to better evaluate possible synergistic benefits of these therapies with re-RT. Marginal recurrence was significantly more frequent in patients who had prior BEV exposure, highlighting the importance of including T2/FLAIR abnormalities into target volumes when safe to do so. Ongoing and future prospective trials are needed to evaluate the best therapeutic algorithm for patients with recurrent glioblastoma. |
| Generalizability | 21 | Discuss the generalizability (external validity) of the study results | Generalizability of our study/data is limited, as this is a retrospective case series, yet insights might be relevant for clinical practice and hypothesis-building. |
| Other information |  |  |  |
| Funding | 22 | Give the source of funding and the role of the funders for the present study and, if applicable, for the original study on which the present article is based | No funding was received explicitly for this project. SMC is on research leave and lead this project during his research fellowship time. In this function, he received support through the “Young Talents in Clinical Research” Beginner’s Grant from the Swiss Academy of Medical Sciences (SAMW) and the Bangerter-Rhyner Foundation. |

Supplementary table 2: Uni- and multivariable Cox regression analysis for OS after re-RT

|  | **UVA** | | **MVA** | |
| --- | --- | --- | --- | --- |
| **Variable** | **p-value** | **HR (95% CI)** | **p-value** | **HR (95% CI)** |
| Age in years at re-RT |  |  |  |  |
| - <58 [median] vs. ≥59 | 0.245 | 1.021 (0.978–1.052) | 0.594 | 1.004 (0.975–1.019) |
| MGMT methylation status |  |  |  |  |
| - Methylated vs. non-methylated^1^ | 0.323 | 0.891 (0.650–1.278) | 0.598 | 0.887 (0.681–1.235) |
| KPS |  |  |  |  |
| - <70 vs. ≥70 | **0.047** | 0.522 (0.276–0.923) | **0.001** | 0.543 (0.281–0.963) |
| Number of recurrences before re-RT |  |  |  |  |
| - 1 vs. ≥2 | **0.005** | 1.430 (0.786–1.671) | 0.214 | 1.276 (0.834–1.587) |
| EQD2 |  |  |  |  |
| - <99.4 [median] vs. ≥99.4 | 0.529 | 1.001 (9.874–1.566) |  |  |
| Lymphocyte count before re-RT |  |  |  |  |
| - <0.96 [median] vs. ≥0.96 | 0.648 | 0.872 (0.598–1.418) |  |  |
| Surgery 6 weeks before re-RT |  |  |  |  |
| - Yes vs. no | 0.678 | 0.675 (0.445–1.391) |  |  |
| Concurrent TMZ |  |  |  |  |
| - Yes vs. no | 0.287 | 1.381 (0.641–2.961) |  |  |
| Concurrent IO |  |  |  |  |
| - Yes vs. no | 0.512 | 0.861 (0.563–1.334) |  |  |
| Prior Bev exposure |  |  |  |  |
| - Yes vs. no | **0.041** | 1.453 (1.091–2.234) | 0.367 | 1.244 (0.673–1.981) |

^1^Non-methylated includes unmethylated and partially methylated.

*Abbreviations:* CI = Confidence interval; HR = Hazard ratio; KPS = Karnofsky performance status; MGMT = MGMT = O^6^-methylguanine-DNA methyltransferase; MVA = Multivariable analysis; Re-RT = Reirradiation; SRS = Stereotactic radiosurgery; SRT = Stereotactic radiotherapy; UVA = Univariable analysis; Vs. = Versus.
